# Continuous Glucose Monitoring Among Patients With Type 1 Diabetes in Rwanda (CAPT1D) Phase I: Prospective Observational Feasibility Study

**DOI:** 10.2196/64585

**Published:** 2025-01-21

**Authors:** Jason Baker, Giacomo Cappon, Jean Claude Habineza, Corey H Basch, Steven Mey, Diana L Malkin-Washeim, Christian Schuetz, Niyonsenga Simon Pierre, Etienne Uwingabire, Alvera Mukamazimpaka, Paul Mbonyi, Sandhya Narayanan

**Affiliations:** 1 Diabetes Empowerment International, New York, NY, United States; 2 Department of Information Engineering, University of Padova, Padova, Italy; 3 Rwanda Diabetes Association, Kigali, Rwanda; 4 School of Health Sciences, University of Rwanda, Kigali, Rwanda; 5 Rwanda Biomedical Center, Kigali, Rwanda

**Keywords:** CGM, feasibility, type 1 diabetes, self-management, complications, Rwanda, time in range, hypoglycemia, continuous glucose monitoring, single-arm, feasibility study, diabetes, diabetes mellitus, euglycemic, HbA1c, observational study, quantitative, adult, health, public health, health informatics

## Abstract

**Background:**

The development of minimally invasive continuous glucose monitoring systems (CGMs) has transformed diabetes management. CGMs have shown clinical significance by improving time in the euglycemic range, decreasing rates of hypoglycemia, and improving hemoglobin A_1c_ (HbA_1c_). In Rwanda, CGMs are currently not routinely used, and no clinical studies of CGM use were identified in the literature.

**Objective:**

This study aims to determine the impact and feasibility of real-time CGM use among people living with type 1 diabetes (T1D) in Rwanda through assessment of sensor use, time in range, rates of hypoglycemia and hyperglycemia, HbA_1c_, and rates of diabetes-related hospitalizations over time.

**Methods:**

The Continuous Glucose Monitoring Among Patients with Type 1 Diabetes in Rwanda (CAPT1D) study is a single-arm, prospective observational study conducted at the Rwandan Diabetes Association clinic in Kigali, Rwanda, aiming to assess the impact and feasibility of CGM use in Rwanda. A cohort of 50 participants diagnosed with T1D were enrolled. Participants were at least 21 years old, undergoing multiple daily insulin therapy, and not currently pregnant. Phase I of the study was conducted over 12 months, using the Dexcom G6 CGM. Phase II and Phase III extended CGM use for an additional 6 months respectively, using the next-generation Dexcom G7 CGM. Here, we report the quantitative results of the Phase I study.

**Results:**

Participants used the sensor for >80% of the time throughout the study period. A significant increase in time in range was observed within 3 months and sustained over 12 months. HbA_1c_ decreased significantly in 3 months and stayed lower throughout the 12-month period. Mean HbA_1c_ levels decreased by 2.8% at 6 months (*P*<.001) and 3.2% at 12 months (*P*<.001). A total of 12 diabetes-related hospitalizations were reported during the study period. No cases of diabetic ketoacidosis or episodes of severe hypoglycemia occurred.

**Conclusions:**

Significant and meaningful improvements in key glycemic indices indicate the potential feasibility and impact of a CGM among people living with T1D in Rwanda. Future studies could be designed to include pre- and postintervention analysis to determine the effectiveness in terms of complications and costs.

## Introduction

The International Diabetes Federation reports that there are 537 million adults aged 20‐79 years living with diabetes worldwide, with estimates that this number will increase to 700 million by 2045 [1]. There are more than 19 million adults with diabetes living in the African region [1], and in the International Diabetes Federation’s latest report, Rwanda had a prevalence of diabetes registered at 2.7% (168,900/6,358,500) [2]. For those people with diabetes classified as having type 1 diabetes (T1D), the only treatment is through the administration of insulin exogenously. In addition, people with type 2 diabetes may need intensive insulin therapy with disease progression or poor management with noninsulin therapies. In both cases, glucose monitoring is necessary [3] to prevent or treat acute (hypoglycemia, hyperglycemia, and diabetic ketoacidosis [DKA]) or chronic (microvacular and macrovascular) complications, and more frequent monitoring has been shown to have better diabetes outcomes [4].

Traditional methods of glucose monitoring among people with diabetes have been through finger-stick blood sugar checks, referred to as self-monitoring blood glucose (SMBG), involving pricking one’s finger with a small needle at different times during the day. The blood drawn is then transferred to a test strip which is read by a blood glucose monitor to display the glucose value at that point in time. There may be several obstacles related to SMBG—an invasive method that can be accompanied by discomfort and inconvenience [5,6] and relies on glucose meters that vary in accuracy [3]. Furthermore, SMBG provides very limited insight into glycemic trends, as only a few data points per day are available for decision-making [6].

In the absence of regular information regarding glucose levels, the primary way to understand how well diabetes is managed is through a blood test known as the hemoglobin A_1c_ (HbA_1c_) test, which measures the percentage of glycated hemoglobin in red blood cells. This is an average over a 3‐ to 4-month period and provides a retrospective evaluation of therapeutic and self-management efficacy [7]. As such, it does not provide meaningful information regarding frequent or sustained high or low blood glucose values. Therefore, it is not the best tool to assist people with diabetes with treatment interventions for daily fluctuations in blood glucose [8].

The development of minimally invasive continuous glucose monitoring systems (CGMs) has transformed diabetes management [9]. CGMs provide continuous readings throughout the day and provide meaningful insight to people with diabetes regarding the time in range (TIR), as well as alerts when the glucose values veer from the range into higher (time above range [TAR]) or lower (time below range [TBR]) levels. These alerts then allow for immediate treatment action, thereby reducing the time spent in the times outside of the euglycemic range [8].

CGMs have shown clinical significance by improving time in the euglycemic range, decreasing rates of hypoglycemia and HbA_1c_ levels [10], and lowering rates of emergency department visits and hospitalizations for hypoglycemia [11]. Improved outcomes have also been shown when a CGM is introduced in the first year after diagnosis with the improvements sustained over time, for as long as 7 years [12].

In Rwanda, CGMs are currently not routinely used, and no clinical studies of CGM use were identified in the literature. The study objectives were to determine the impact and feasibility of real-time CGM use among people living with T1D in Rwanda through assessment of sensor use; TIR; HbA_1c_; and rates of hypoglycemia, diabetes-related hospitalizations (DRHs), and DKA.

## Methods

### Study Design

The Continuous Glucose Monitoring Among Patients with Type 1 Diabetes in Rwanda (CAPT1D) study is a single-arm, prospective observational study conducted at the Rwandan Diabetes Association (RDA) clinic in Kigali, Rwanda, aiming to assess the impact and feasibility of CGM use in Rwanda.

The study protocol involved a run-in phase with blinded CGM (Dexcom G6, Dexcom Inc), during which participants received instructions in August 2022. Subsequently, continuous glucose monitoring was unblinded, and participants underwent a 12-month period during which they visited the RDA clinic every 20 days to upload CGM data onto the Dexcom Clarity (Dexcom Inc) platform. Participants were queried for and self-reported episodes of DKA (date, symptoms, treatment), instances of hypoglycemia, and DRHs at each study visit.

### Study Sample and Recruitment

A convenience sample of established patients of the RDA was selected using a 2-stage process. First, the RDA study staff created a list of patients who met the inclusion criteria, as follows: older than 21 years, not pregnant, and treated with multiple daily doses of insulin. Second, this list was polled to identify those participants who were willing and able to commute to the RDA clinic as frequently as needed for orientation, to change sensors, and to upload data. Individuals were informed prior to enrollment that they would not be paid to participate in the study, but that transportation costs to and from the clinic will be reimbursed. The first 50 participants who agreed to participate were then enrolled in the study. The sample size was determined by the capacity of the staff and the availability of equipment. Phase I of the study was conducted over 12 months, using the Dexcom G6 CGM. Phase II and Phase III extended CGM use for an additional 6 months respectively, using the next-generation Dexcom G7 CGM. The number of participants was reduced to 47 when 3 participants withdrew from the study at month 6. Thereafter, the study sample was further reduced to 42 as a result of 5 participants joining another study where they received additional education possibly biasing the results of CAPT1D. To handle the fact that several patients were removed from month 6 on, statistical analysis was adjusted with the last observation carried forward method, that is, the last available measurement for a patient is carried forward to later time points. Here we report the quantitative results of the Phase I study.

### Ethical Considerations

Ethical approval was obtained from the University of Rwanda School of Medicine Ethical Committee, and the study was registered with the institutional review board under number #271/CMHSIRB/2022. Participants were provided with all details regarding the study’s objectives, procedures, potential risks, and benefits before requesting consent. At this time, any questions were addressed, and privacy and confidentiality policies were explained. A consent form outlining the study’s scope, potential risks, the voluntary nature of participation, and the right to withdraw at any time was provided and signed by each participant. Additionally, consent to use photographs and other recorded material in published material was obtained. To this end, all data collected for this study have been fully anonymized and deidentified to ensure the privacy and confidentiality of all participants, and to prevent linkage of the data to individual participants. Participants were compensated varied amounts for their transportation to and from the clinic, provided light snacks and beverages during clinic visits, and received the CGMs for no cost. There is no identification of individual participants/users in any aspect of this manuscript.

### Statistical Methods

CGM data were preprocessed and analyzed using AGATA [13], an open-source software tool that computes glucose control metrics in accordance with the definitions of the International Consensus on CGM-derived metrics for clinical trials [14]. Initially, CGM data were preprocessed by aligning each data point on a uniform 5-minute time grid and linearly interpolating data gaps up to 30 minutes.

Subsequently, AGATA was used to extract a set of well-established metrics [14] quantifying glucose control, including 5 metrics for glucose variability (mean, SD, coefficient of variation, glucose management indicator, and glucose risk index); 6 metrics describing the percentage of time spent in specific glucose ranges (level 1 and level 2 TBR [TBR_L1_ and TBR_L2_], defined as <70 mg/dL and <55 mg/dL respectively; level 1 and level 2 TAR [TAR_L1_ and TAR_L2_], defined as >180 mg/dL and >250 mg/dL, respectively; TIR, defined as 70-180 mg/dL; and time in tight range [TITR], defined as 70-140 mg/dL); and a metric quantifying CGM use, namely the percentage of time the CGM was active in the considered period (%CGM_USE_).

To align CGM data to HbA_1c_ samples collected every 3 months, each metric has been calculated by aggregating CGM data over 3-month periods and compared with the baseline computed during the initial run-in session at month 0.

Hereafter, for the sake of brevity, we will label M0 the baseline run-in session, while we will use M3, M6, M9, and M12 to represent the metric values computed using data from M0 to month 3, month 3 to month 6, and so forth.

Results were presented as the mean (SD) if the metric distribution followed a Gaussian distribution (determined by the Lillefors test at a significance level of 5%), or as the median (IQR) if not. Changes were evaluated by comparing M0 versus M6, M6 versus M12, and M0 versus M12 using a 2-tailed *t* test with a significance level of 5% for Gaussian-distributed metrics. Alternatively, the Wilcoxon rank sum test with a significance level of 5% was used.

## Results

**Figure 1.**
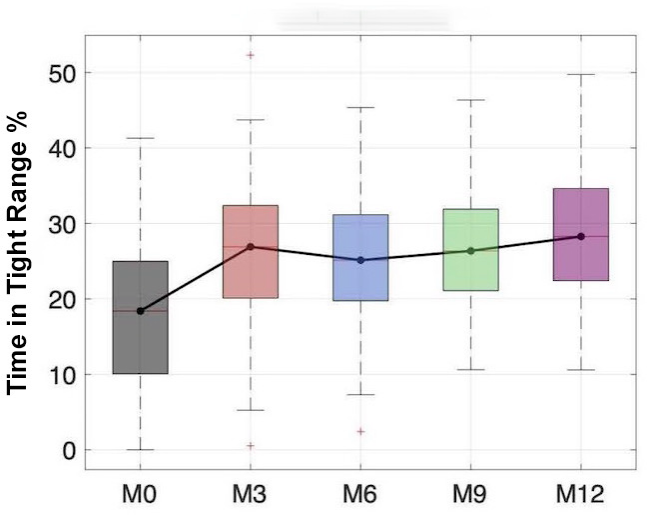
Box plot distributions of time in tight range were obtained at M0 (gray), M3 (red), M6 (light blue), M9 (green), and M12 (violet) from the Continuous Glucose Monitoring Among Patients with Type 1 Diabetes in Rwanda study, a single-arm, prospective observational study conducted at the Rwandan Diabetes Association clinic in Kigali, Rwanda (2022‐2023). Median values are highlighted and connected by a black solid line.

**Figure 2.**
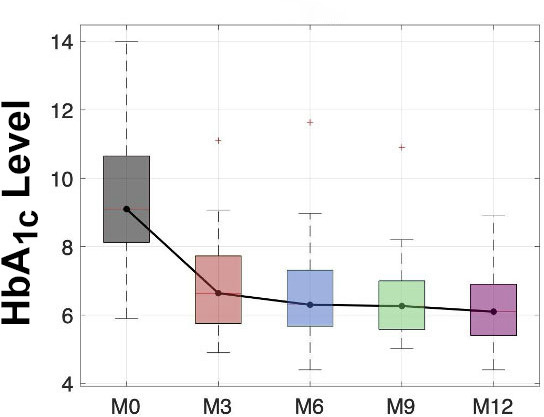
Box plot distributions of HbA_1c_ were obtained at M0 (gray), M3 (red), M6 (light blue), M9 (green), and M12 (violet) from the Continuous Glucose Monitoring Among Patients with Type 1 Diabetes in Rwanda study, a single-arm, prospective observational study conducted at the Rwandan Diabetes Association clinic in Kigali, Rwanda (2022‐2023). Median values are highlighted and connected by a black solid line. HbA_1c_: hemoglobin A_1c_.

The results are illustrated in Table 1. Participants used the sensor for >80% of the time throughout the study period. TIR was defined as 70‐180 mg/dL. The absolute change from baseline to 6 months for TIR was an increase of 9.9% (*P*<.001), and from baseline to 12 months, the absolute change was an increase of 14.7% (*P*<.001). Thus, the significant improvement in TIR at month 6 was maintained (indeed increased) at 12 months. TITR was defined as 70‐140 mg/dL. The absolute change from baseline to 6 months for TITR was an increase of 7.1%, and that from baseline to 12 months was an increase of 10.4% (*P*<.001) (Figure 1). Time in hypoglycemia or TBR was defined as <70 mg/dL. From baseline to 6 months, this increased by 0.7%, which was not statistically significant (*P*=.31), and from baseline to 12 months, there was an increase of 2.5% (*P*<.001). The TAR is defined as >180 mg/dL. The absolute change from baseline to 6 months for TAR was a decrease of 9.7% (*P*<.01), and that from baseline to 12 months was a decrease of 15.3% (*P*<.001). Mean HbA_1c_ levels decreased by 2.8% at 6 months (*P*<.001) and 3.2% at 12 months (*P*<.001) (Figure 2). A total of 12 DRHs were reported during the study period. More detailed analysis of these hospitalizations is underway. No cases of DKA or episodes of severe hypoglycemia occurred.

**Table 1.** Glycemic indicators at baseline and at 3, 6, 9, and 12 months from the Continuous Glucose Monitoring Among Patients with Type 1 Diabetes in Rwanda study, a single-arm, prospective observational study conducted at the Rwandan Diabetes Association clinic in Kigali, Rwanda (2022‐2023).

Metrics	Baseline (N=46)		M3 (N=47)		M6 (N=47)		M9 (N=42)		M12 (N=42)		*P* value (baseline vs M12)	*P* value (baseline vs M6)	*P* value (M6 vs M12)
	Mean (SD)	Median (IQR)	Mean (SD)	Median (IQR)	Mean (SD)	Median (IQR)	Mean (SD)	Median (IQR)	Mean (SD)	Median (IQR)			
Percentage of sensor data	—^a^	99.8941 (98.7353‐100)	—	84.4136 (77.8819‐91.8981)	—	83.8927 (73.1684‐93.2301)	—	86.5239 (81.3619‐95.2238)	—	88.0305 (73.5031‐95.7562)	<.001	<.001	.49
Time in range (%)	30.9998 (15.0695)	—	42.4303 (14.683)	—	40.8986 (12.8338)	—	44.0248 (12.4312)	—	45.6591 (12.0551)	—	<.001	<.001	.08
Time in tight range (%)	18.098 (9.5095)	—	25.8216 (10.056)	—	25.2415 (8.7807)	—	27.1875 (8.7808)	—	28.4829 (8.6731)	—	<.001	<.001	.08
Time below range (%)	—	2.369 (0.1768‐6.4643)	—	2.146 (1.2232‐6.1304)	—	3.0203 (1.054‐6.4066)	—	2.9182 (1.2722‐7.7068)	—	4.8789 (1.602‐8.9319)	<.001	.31	.17
Time above range (%)	64.1901 (17.409)	—	53.5695 (16.3889)	—	54.5215 (14.4949)	—	50.9759 (13.8959)	—	48.8406 (13.6259)	—	<.001	<.001	.06
Mean glucose (mg/dL)	238.9856 (53.6842)	—	—	193.5024 (181.2758‐232.7176)	209.5659 (40.2107)	—	200.1775 (34.7531)	—	194.077 (32.4736)	—	<.001	<.001	.05
Glucose management indicator	9.0265 (1.2841)	—	—	7.9386 (7.6461‐8.8766)	8.3228 (0.96184)		8.0982 (0.83129)	—	7.9523 (0.77677)	—	<.001	<.001	.05
Glucose risk index	—	99.5992 (76.2825‐100)	75.5983 (19.3713)	—	78.7051 (17.9064)	—	—	81.6141 (57.678‐90.9717)	73.6757 (19.2475)	—	<.001	<.001	.21
Coefficient of variation	41.107 (10.4761)	—	42.7715 (7.7309)	—	43.8174 (7.1405)	—	44.2991 (6.5068)	—	44.6247 (6.6217)	—	.07	.15	.58
SD (mg/dL)	94.2823 (19.0105)	—	87.6943 (17.0699)	—	90.4366 (15.6548)	—	88.2446 (17.5455)	—	86.2282 (17.1804)	—	<.001	.29	.23
Hypoglycemic events per week (n)	—	4.4336 (0.70961‐9.1829)	—	4.6668 (1.9445‐7.2531)	—	4.978 (2.3529‐7.7781)	—	4.9002 (2.6445‐9.1781)	6.4021 (4.3053)	—	.14	.50	.38
Hyperglycemic events per week (n)	16.2707 (4.8316)	—	16.5623 (4.4013)	—	16.5226 (4.8881)	—	17.6488 (4.882)	—	16.7118 (5.7817)	—	.70	.80	.87

a Not applicable.

## Discussion

The findings from Phase I of the CGM use among individuals with T1D in Rwanda provide insight into the impact and feasibility of real-time CGM use. As noted previously, the key objectives of this study included evaluating sensor use; TIR; HbA_1c_ levels; and rates of hypoglycemia, DRH, and DKA. Study participants demonstrated high levels of sensor use (80% of the study period). As noted in the results, TIR improved with increased levels at 6 and 12 months. HbA_1c_ levels also improved with marked decreases at 6 and 12 months. Of note, there was a mild, nonstatistically significant increase in TBR, which is indicative of hypoglycemia, but there was also a statistically significant reduction in TAR at 6 and 12 months. There was no report of severe hypoglycemia or DKA events, and only 12 DRHs were reported during the study. In sum, these data indicate a low level of adverse effects.

The low attrition rate, the high percentage of sensor use, and the willingness of participants to return to the clinic every 20 days for data uploads indicated the feasibility of continuous glucose monitoring in Rwanda among patients with T1D. The findings from this study augment those from other studies conducted in African countries showing that continuous glucose monitoring is a feasible alternative to SMBG [15,16].

In a study aiming to assess the feasibility of CGM use in Malawi, one of the challenges identified was the inability of participants to change their own sensors [16]. However, in this study, participants were given sufficient training so that they could change their sensors on their own. Additionally, participants visited the clinic frequently per study protocol, providing ample opportunity for study staff to help provide guidance on sensor removal and insertion.

A systematic review of 22 randomized controlled trials comparing continuous glucose monitoring and SMBG found a significant reduction in HbA_1c_ with the CGM group [17]. Although this study was not interventional, the observed results showed a similar significant reduction in HbA_1c_ with the use of a CGM.

Further, the findings from this study are aligned with other studies showing a reduction in HbA_1c_ with the use of a CGM [10,18] and consistent with prior studies showing that the use of a CGM increased TIR, a desirable outcome for people with diabetes[18].

There were several limitations of this study that limited validity and generalizability. The single-arm observational design lacks a control group, which is consistent with a feasibility study but nonetheless limits between-group comparisons, the ability to isolate the effect of CGM use, the ability to conduct complete comparison analyses, and the ability to generalize findings.

While we were able to glean insight from this convenience sample, the sample was small at the outset, and the further reduction due to attrition impacts statistical power and effect size. Our use of the last observation carried forward method for missing data could potentially lead to bias.

As with any study that uses self-reported information, bias can be introduced and accuracy can be compromised. Notwithstanding these limitations, the length of this study, conducted over a 1-year period, provided a meaningful amount of data points for analysis. These preliminary results can serve as a foundation for future studies that can include control groups and interventions. This work is particularly relevant given the dearth of studies to date related to CGM use in people with T1D in Rwanda.

The results suggest that implementing CGM technology in T1D patients in Rwanda is a feasible and promising method for improving glycemic outcomes among people with T1D. Within 3 months, results indicate improvements in glycemic management, with the improvements sustained over the 1-year study period, as evidenced by better TIR, TITR, and TAR and lower HbA_1c_ levels. These outcomes indicate potential for better diabetes self-management, thereby impacting rates of diabetes-related complications and their associated personal and social costs. Results from this study can be used to conduct more rigorous interventional studies to confirm the beneficial impact of a CGM on health outcomes for people with diabetes.

## Abbreviations

CAPT1D: Continuous Glucose Monitoring Among Patients with Type 1 Diabetes in Rwanda
CGM: continuous glucose monitoring system
DKA: diabetic ketoacidosis
DRH: diabetes-related hospitalization
HbA_1c_: hemoglobin A_1c_
RDA: Rwandan Diabetes Association
SMBG: self-monitoring blood glucose
T1D: type 1 diabetes
TAR: time above range
TBR: time below range
TIR: time in range
TITR: time in tight range
